# Hypoxia as a Target for Combination with Transarterial Chemoembolization in Hepatocellular Carcinoma

**DOI:** 10.3390/ph17081057

**Published:** 2024-08-11

**Authors:** Zizhuo Wang, Qing Li, Bin Liang

**Affiliations:** 1 Hubei Key Laboratory of Molecular Imaging, Department of Radiology, Union Hospital, Tongji Medical College, Huazhong University of Science and Technology, 1277 Jiefang Road, Wuhan 430022, China; wzz199902@163.com; 2 Department of Radiology, The First Affiliated Hospital of Soochow University, Suzhou 215006, China; 20224032007@stu.suda.edu.cn

**Keywords:** hypoxia, tumor microenvironment, hypoxia-inducible factor, hepatocellular carcinoma, transarterial chemoembolization

## Abstract

Hypoxia is a hallmark of solid tumors, including hepatocellular carcinoma (HCC). Hypoxia has proven to be involved in multiple tumor biological processes and associated with malignant progression and resistance to therapy. Transarterial chemoembolization (TACE) is a well-established locoregional therapy for patients with unresectable HCC. However, TACE-induced hypoxia regulates tumor angiogenesis, energy metabolism, epithelial-mesenchymal transition (EMT), and immune processes through hypoxia-inducible factor 1 (HIF-1), which may have adverse effects on the therapeutic efficacy of TACE. Hypoxia has emerged as a promising target for combination with TACE in the treatment of HCC. This review summarizes the impact of hypoxia on HCC tumor biology and the adverse effects of TACE-induced hypoxia on its therapeutic efficacy, highlighting the therapeutic potential of hypoxia-targeted therapy in combination with TACE for HCC.

## 1. Introduction

Hepatocellular carcinoma (HCC) is one of the most common malignancies and a leading cause of cancer deaths worldwide [1]. Based on the Barcelona-Clinic Liver Cancer (BCLC) staging system, HCC is divided into very early, early, intermediate, advanced, and terminal stages. Accordingly, current treatment options for HCC include liver resection, liver transplantation, ablation, transarterial chemoembolization (TACE), systemic therapies, and best supportive care [2]. The former three therapies, as potentially curative treatments, are only suitable for the very early and early stages. However, the majority of HCC patients, particularly in China, are diagnosed at the intermediate or advanced stages, and TACE and systemic therapies have been the mainstays of treatment for HCC [3,4].

TACE is a well-established locoregional therapy for patients with unresectable HCC. The concept of TACE is to induce a comprehensive effect of cytotoxicity and ischemia through the intraarterial infusion of chemotherapeutic agents, followed by the embolization of tumor-feeding arteries [5]. However, recent studies have shed light on the implications of TACE-induced hypoxia in liver tumors. Hypoxia, as an integral characteristic of solid tumors, has proven to be involved in multiple tumor biological processes and associated with malignant progression and resistance to conventional chemotherapy and radiotherapy [6]. Similar results were reported in HCC after TACE. The hypoxic microenvironment induced by TACE results in the activation of hypoxia-inducible factors (HIFs) and the overexpression of vascular endothelial growth factor (VEGF) in residual tumors [7,8,9,10,11,12,13]. Accordingly, anti-angiogenic therapy has been combined with TACE to treat HCC [14]. However, given the fact that angiogenesis is one of the tumor’s biological responses to hypoxia, direct hypoxia-targeted therapy may represent a more effective strategy than anti-angiogenic therapy for HCC. In this review, we describe the relationship between hypoxia and HCC, with a focus on the implications of TACE-induced hypoxia for efficacy and the therapeutic potential of the combination of TACE with hypoxia-targeted therapy against HCC.

## 2. The Role of Hypoxia in HCC Biology

Hypoxia is an integral characteristic of most solid tumors, including HCC [15,16]. Much has been learned regarding the molecular pathways of hypoxia and the impact of hypoxia on HCC biology. HIFs, and the HIF-1 transcription factor in particular, regulate the cellular response to hypoxia. By functionally interacting with other transcription factors, HIF-1 activates the transcription of many target genes that code for proteins that are involved in angiogenesis, glucose metabolism, cell survival/proliferation, and invasion/metastasis, thereby triggering a series of biological reactions [16]. This can contribute to the development and progression of HCC. Firstly, hypoxia contributes to hepatocarcinogenesis [17]. Chronic hepatitis and cirrhosis cause fibrosis, which disrupts the normal vascular system, reduces hepatic blood supply, and thus leads to hypoxia [18]. Hypoxia and HIF-1 enhance the stemness of HCC cells and promote carcinogenesis [19]. Hypoxia can also induce hexokinase II [20] and insulin-like growth factor (IGF)-2 [21] to stimulate HCC growth and myeloid cytokine-1 [22] to impede HCC apoptosis. Secondly, a hypoxic environment induces angiogenesis in cirrhotic and HCC tissues [18]. Under hypoxia, HIF-1 is activated and induces the production of angiogenic factors [23], such as VEGF [24], basic fibroblast growth factor (bFGF) [24], and IGF-2 [21,25]. Although these factors promote tumor angiogenesis [18], the newly formed blood vessels fail to alleviate the hypoxic environment due to their disorganization and distortion [26,27]. Thirdly, hypoxia influences abnormal glucose metabolism in cancer cells, promoting the Warburg effect [28], where cells prefer glycolysis to mitochondrial ATP synthesis even in the presence of oxygen [29]. HIFs drive this metabolic shift by upregulating glycolytic enzymes such as pyruvate dehydrogenase kinase 1 (PDK1) and lactate dehydrogenase A (LDHA) [30,31,32,33]. This metabolic adaptation supports the energy needs of hypoxic cancer cells, sustaining their growth and survival [34]. Fourthly, hypoxia promotes metastasis through the HIF-1 pathway [35], which regulates the expression of genes involved in HCC invasion and metastasis [36,37,38]. Hypoxia contributes to tumor cell detachment, adhesion, and migration by downregulating the expression of epithelial cadherin (E-cadherin) [39,40], upregulating the expression of integrin genes [41], stimulating the release of matrix metalloproteinase (MMP)-2 [42] and the urokinase plasminogen activator (uPAR) [43], and inducing the production of autocrine motility factors (AMFs) such as hepatocyte growth factor (HGF) [43,44,45]. Fifthly, hypoxia is related to resistance to radiotherapy and chemotherapy by regulating multiple cellular adaptive responses and gene expressions [46,47,48], and by inducing cell cycle arrest or retardation [49,50,51]. Finally, hypoxia significantly affects the immune response by maintaining an immunosuppressive tumor microenvironment (TME) [52,53]. Hypoxia-induced HIF impairs the function or infiltration of immune cells and further prevents the activation of immune effector cells by upregulating complex regulatory molecules [54,55]. Hypoxia also recruits immunosuppressive cells to block the immune response [52,56]. These processes promote the immune escape of tumor cells and lead to drug resistance in anti-tumor immunotherapy. Taken together, hypoxia forms a complex network in HCC, playing a comprehensive and critical role in hepatocarcinogenesis, angiogenesis, abnormal glucose metabolism, metastasis, drug resistance, and immunosuppression.

## 3. TACE of HCC

TACE is the recommended first-line therapy for patients with intermediate-stage disease, and is, by far, the most common technique used to treat unresectable HCC [2,5]. The rationale for TACE comes from previous findings that normal hepatic tissue receives most of its blood supply from the portal vein, whereas hepatic malignancies receive most of their blood supply from the hepatic artery [57]. Therefore, it is reasonable to employ the hepatic artery as an approach to target the tumor while preserving the normal liver tissue. TACE treatment consists of the transcatheter intraarterial delivery of chemotherapy combined with embolization of the tumor-feeding arteries, which result in the comprehensive effects of cytotoxicity and ischemia against the tumor [5].

TACE can be technically divided into conventional TACE and drug-eluting beads TACE (DEB-TACE). Conventional TACE involves the infusion of single or multiple chemotherapeutic agents with or without ethiodized oil, followed by embolization with particles such as gelatin sponge, polyvinyl alcohol, or calibrated microspheres [58]. In this way, high concentrations of chemotherapeutic agents can be directly delivered to the tumor bed. Additionally, embolization of the tumor-feeding arteries can not only induce tumor ischemic and hypoxic necrosis but also enhance the cytotoxic effect of chemotherapy by reducing drug washout, prolonging the interaction time between drugs and tumor cells, and improving drug penetration within the tumor [59]. Previously, two randomized controlled trials demonstrated that conventional TACE resulted in higher overall survival compared to the best supportive care [60,61]. The therapeutic efficacy of conventional TACE on HCC has been confirmed by subsequent clinical studies [62].

In contrast, DEB-TACE is defined as the administration of calibrated microspheres onto which chemotherapeutic medication is loaded or adsorbed with the intention of sustained in vivo drug release [63]. Drug-eluting microspheres have the ability to load chemotherapeutic agents (e.g., doxorubicin, epirubicin, and idarubicin) and release them in a controlled and sustained mode. Accordingly, DEB-TACE allows higher doses of chemotherapy with lower systemic exposure, along with permanent embolization. In a randomized phase II trial, DEB-TACE yielded an improved radiologic tumor response and toxicity compared to conventional TACE [64]. However, the superiority of DEB-TACE over conventional TACE has never been demonstrated in terms of overall survival (OS) in clinical trials [65,66,67,68]. Therefore, there is currently insufficient evidence to recommend DEB-TACE over conventional TACE. Developing novel microspheres with superior drug-loading mechanisms and loading other drugs (e.g., drugs targeting hypoxic cells) or formulations (e.g., oncolytic viruses and immunostimulants) will be the focus of future research in the DEB-TACE field.

## 4. TAE-Induced Hypoxia and Its Implications for Tumor Biological Processes

Transarterial embolization (TAE) is an important component of TACE procedures. However, recent studies have raised questions concerning the precise effect of embolization on liver tumors. Research has found that diversity in the degree and duration of hypoxia may have different effects on tumor cells. Severe or sustained hypoxia induces cell death, whereas mild or transient hypoxia may lead to a series of adaptive responses in tumor cells, such as activating signaling pathways that regulate cell survival, glucose metabolism conversion, angiogenesis, infiltration and metastasis, and drug resistance, thereby allowing the tumor cells to survive or even evolve [26]. Theoretically, the embolization of tumor-feeding arteries results in ischemia of the tumor and subsequent tumor necrosis. Unfortunately, due to the complexity of the blood supply for HCC and the limitations of interventional embolization techniques, embolization of the tumor-feeding vessels may be incomplete. This may result in part of the tumor continuing to survive and even evolving in a hypoxic microenvironment because of the reduced blood supply.

Central to these processes is the role of HIF-1α. Previous research has indicated a notable increase in HIF-1α, VEGF, hexokinase II, cyclooxygenase-2 (COX-2), and programmed death-ligand 1 (PD-L1) levels in tumors following TAE [7,13,69,70,71,72]. In two previous animal studies, elevated expression of HIF-1α was reported in liver tumors after TAE [69,73]. A subsequent animal study used a modified Clark-type microelectrode research system to measure *pO*_2_ and found that TAE rapidly reduced tumor oxygenation [8]. The study also found that positive HIF-1α staining was detected predominately in viable tumor cells in the tumor peripheral zone, which displayed a distribution pattern similar to that observed in hypoxic areas marked by pimonidazole [8]. These studies suggest that the TAE of liver tumors resulted in HIF-1α overexpression as a result of intratumoral hypoxia generated by the procedure.

Two previous clinical studies measured VEGF levels in serum and plasma in HCC patients and found that the VEGF levels increased significantly after TAE [9,10]. Similar results were observed in another clinical study comparing VEGF expression in tumor specimens between HCC patients pretreated with TACE and without TACE [11]. Subsequent animal studies confirmed that TAE-induced hypoxia resulted in increased VEGF expression, promoting the neovascularization of residual tumors [12,13]. Another initial clinical study found that the expression of hexokinase II mRNA was increased in tumor tissue in some HCC patients pretreated with TAE, and hexokinase II mRNA expression was significantly correlated with HIF-1α protein expression. In addition, both HIF-1α and hexokinase II protein expressions were co-localized in the cancer cells adjacent to necrotic areas. This study suggests that HCC may switch the metabolic profile to glycolysis through HIF-1α [7]. In addition, a recent clinical study demonstrated the upregulation of HIF-1α and COX-2 proteins together with epithelial-to-mesenchymal transition (EMT) alteration in HCC tissues following TACE treatment, which was associated with a negative correlation with overall survival [71]. Also, a preclinical study demonstrated that hypoxia selectively upregulated PD-L1 on myeloid-derived suppressor cells (MDSCs) via HIF-1α. Blocking PD-L1 under hypoxia enhanced MDSC-mediated T cell activation by modulating MDSC cytokine production of interleukin 6 (IL-6) and interleukin 10 (IL-10) [72]. Taken together, these results suggest that hypoxia after TAE of liver tumors is involved in tumor angiogenesis, energy metabolism, EMT, and immune processes, which may have adverse effects on the therapeutic efficacy of TAE.

## 5. Hypoxia-Targeted Therapy for HCC

Tumor hypoxia has emerged as an attractive therapeutic area due to its essential role in cancer. However, extensive basic research and clinical trials are still required to validate its potential therapeutic value. Based on the therapeutic mechanism of action, four general strategies were developed in the past 20 years.

### 5.1. Targeting HIF and HIF-Related Hypoxia Signaling

HIF-1α/HIF-2α inhibitors can be categorized as indirect or direct. Indirect HIF inhibitors regulate upstream and downstream effectors in the HIF pathway, while direct inhibitors decrease HIF mRNA expression, protein synthesis, or DNA binding. Several recent HIF inhibitors that show promising potential in treating HCC, such as RO7070179 (EZN-2968) [74], CT-707 [75], PT-2385 [76], meloxicam [77], and various natural agents [78,79,80,81,82] (e.g., camptothecin analogs, curcumin, sanguinarine, resveratrol, ginsenosides). Additionally, in 2021, belzutifan (PT2977) was approved by the U.S. Food and Drug Administration (FDA) for renal cell carcinoma (RCC) and other tumors in patients with von Hippel-Lindau syndrome [83,84], making it the first selective HIF-2 inhibitor to receive approval. However, its efficacy in HCC remains to be determined.

### 5.2. Prodrugs Activated by Hypoxia

Hypoxia-activated prodrugs (HAPs) are inactive compounds that are converted into active drugs via enzymatic or metabolic processes under hypoxic conditions, especially hypoxic tumor cells within the body [85]. HAPs may be able to bypass drug resistance mechanisms that are commonly associated with traditional chemotherapy while minimizing damage to healthy tissue [85,86]. Several bioreductive prodrugs have reached clinical trials for the treatment of HCC, such as tirapazamine (TPZ) [87], TH-302 (evofosfamide) [88], CEN-209 (SN30000), Myo-inositol trispyrophosphate (ITPP) [89], and PR-104 [90].

### 5.3. Hypoxia-Selective Gene Therapy

Antisense gene therapy targeting HIF-1, as one of the gene therapies, refers to constructing recombinant plasmids of antisense HIF-1α, transferring these plasmids into hypoxic cells, and transcribing antisense RNA to exert the inhibitory effect of HIF-1α [91]. Oncolytic adenovirus (Ovs) that targets hypoxic tumors selectively infects tumor cells by utilizing internal gene mutation or the metabolic reprogramming of tumor cells and then replicates tumor cells to kill target cells or to indirectly kill tumors by stimulating the immune system’s antitumor response [92,93]. In addition, genetic engineering approaches can be used to construct high-affinity NK (haNK) cells, which can improve tolerance against acute hypoxia and maintain the functions of NK cells to kill cancer cells [94]. Hypoxia-directed enzyme prodrug gene therapy uses anaerobes to transfer functional genes to the tumor hypoxia zone [95]. However, current studies have shown that the HIF-specific strategy of using hypoxia-selective gene therapy is controversial.

### 5.4. Target Other Hypoxia-Associated Biological Processes and Pathways

Hypoxia-induced effects are multifaceted, and the molecular events underlying the adaptive response to hypoxia in HCC involve an intricate interplay. The exploration of VEGF signaling is a well-established and widely utilized field of study [96]. Various angiogenesis inhibitors that suppress VEGF have demonstrated efficacy for HCC [97,98,99,100]. Currently, combination therapies with VEGF inhibitors are being extensively investigated in clinical trials [101,102,103]. In addition, a novel crosstalk between inflammatory and hypoxic TME was revealed to be associated with the PI3K/AKT/mTOR pathway [104], which can be regulated by ruscogenin to reduce the expression of VEGF and HIF-1α, among others, and thus inhibit HCC metastasis [105]. Numerous other biological processes and pathways associated with hypoxia, such as biotherapy targeting anoxic tumor bacteria [106,107] and metabolic dysregulation related to hypoxia [108,109], have also been investigated for treating HCC. However, their feasibility and true efficacy still need to be further explored.

## 6. Combination of Hypoxia-Targeted Therapy and TACE for HCC

While hypoxia-targeted treatment has demonstrated potential in vitro studies, its effectiveness in clinical trials is frequently constrained. The researchers suggest that this limitation may stem from the heterogeneity in tumor types and levels of hypoxia within the tumor. It is hypothesized that combining hypoxic tumor-targeting drugs with interventional embolization could lead to mutually beneficial and synergistic anti-tumor outcomes in the treatment of liver cancer. This section aims to summarize the available drugs or pathways that target hypoxic tumor cells in combination with TACE for the treatment of HCC. The combination of hypoxia-targeted therapy and TACE for HCC is summarized in Table 1, Table 2 and Table 3.

### 6.1. Combination of HIF-Related Pathway Inhibitors and TACE

#### 6.1.1. HIF-1

Melatonin (MLT) is an endogenous hormone secreted by the pineal gland [110]. The anti-tumor mechanism of MLT is primarily to inhibit tumor angiogenesis by inhibiting the HIF-1α/VEGF signaling pathway [111,112]. As early as 2002, TACE, in combination with MLT, was used to treat patients with advanced primary HCC [113]. The results demonstrated that MLT reduced hepatic impairment following TACE and enhanced the immune activity of patients. Recently, MLT was loaded on a temperature-sensitive nano gel, p(N-isopropyl-acrylamide-co-butyl methylacrylate) (PIB-M), for tumor embolism in VX2 rabbit models by Chen et al. [114]. The results of the study confirm that MLT can inhibit the growth and migration of HepG2 and LM3 cells by targeting HIF-1α, MMP-2, MMP-9, and E-cadherin in vitro. Furthermore, the concentration of MLT in the tumor after embolization remains at a high level for the following three days, which suggests that this sustained effect on the tumor cells and TME may be achieved.

A number of natural compounds have been demonstrated to inhibit HIF-1α and VEGF. A combination with TACE has been shown to have promising evidence of targeting hypoxia, indicating potential for clinical translation in the treatment of hepatocellular carcinoma. Furthermore, 10-Hydroxycamptothecin (HCPT), a camptothecin analog, is a naturally occurring alkaloid with antitumor activity. Camptothecin analogs can mediate S-phase cytotoxic effects through the induction of a stable DNA topoisomerase I (Topo I) complex, which can lead to DNA linkage breaks. The ability to inhibit HIF-1 has also been found. Our previous work [115] found that the levels of HIF-1, VEGF, and microvessel density (MVD) were comparable following the intrahepatic arterial infusion of distilled water and HCPT+TACE (*p* > 0.05), indicating that HCPT significantly inhibits the expression of HIF-1 and angiogenesis in postembolization hepatic tumors. This finding suggests that HCPT may have a broader application in this clinical setting. Curcumin is a highly polyphenolic molecule that has been reported to inhibit the viability of cancer cells and is commonly used to prevent or treat a variety of diseases. Dai et al. [116] observed that the levels of tumor HIF-1α, VEGF, and MVD were significantly reduced in the liposomal curcumin combined with TAE in their initial study. Further investigations [117] revealed that, in addition to the aforementioned findings, curcumin liposome was capable of inhibiting survivin levels, significantly inhibiting cell viability, and promoting apoptosis in the G1 phase by regulating apoptosis-related molecules. The ginsenoside Rg3 was found to inhibit the nuclear localization of HIF-1α by binding to HIF-1α [118]. Ginsenoside Rg3 in combination with TAE has been shown to significantly decrease CD31, VEGF expression, and levels of the anti-apoptotic Bcl-2 at both mRNA and protein levels, while significantly increasing pro-apoptotic gene caspase-3 and Bax expression in VX2 rabbit tumor models [119]. A subsequent prospective controlled clinical trial [120] demonstrated that the combination of ginsenoside Rg3 and TACE provided a greater survival benefit than TACE alone in patients with HCC. Additionally, the trial indicated that Rg3 alleviated some of the adverse effects and blood anomalies associated with TACE.

Several other anticancer drugs have also been found to improve the efficacy of TACE by influencing hypoxia signaling, either directly or indirectly. Repeated TACE-induced hepatic hypoxia was found to exacerbate the progression of fibrosis in peritumoral liver tissue, which was associated with the increased expression of carbon tetrachloride, HIF-1α, transforming growth factor-β1 (TGF-β1), and VEGF. The progression of fibrosis and the deterioration of liver function subsequent to TACE may be mitigated and slowed by the HIF-1α inhibitor LW6 [121]. The antitumor effects of arsenic trioxide (ATO)-loaded CSM-TACE have been investigated in VX2 HCC rabbit models. The results indicated that the expression of HIF-1α, VEGF, twist, N-calmodulin, waveform protein, and MMP-9 was decreased in the combined treatment group, while the expression of E-calmodulin was increased [122]. In models of a VX2 liver xenograft tumor, the combination of rapamycin with TACE has also been demonstrated to exhibit anti-tumor neovascularization activity and to inhibit the expression levels of iNOS, HIF-1α, VEGF, Bcl-2, and Bax. The arterial infusion of rapamycin was found to be more effective than intravenous injection, and large doses were observed to present better efficacy [123]. In addition, AMD3100, a chemokine (C-X-C motif) receptor 4 (CXCR4) antagonist, was shown to enhance the therapeutic efficacy of TACE (doxorubicinlipiodol emulsion) in rats with HCC. AMD3100 was found to reduce TACE-induced MVD in HCC tissues by decreasing the expression of HIF-1α and VEGF. Furthermore, it has been shown to promote apoptosis and reduce cell proliferation in HCC [124].

#### 6.1.2. HIF-2

In the context of HIF, HIF-1α is primarily implicated in the acute hypoxic process of the tumor. In contrast, HIF-2α is actively involved in the chronic hypoxic process and exhibits a stronger prognostic correlation with TACE relative to HIF-1α [125]. Furthermore, it has been demonstrated that sorafenib downregulates HIF-1α expression, shifting the hypoxic response from the HIF-1α- to the HIF-2α-dependent pathway. This results in the up-regulation of HIF-2α, which renders hypoxic HCC cells insensitive to sorafenib and induces the expression of VEGF and cyclin D1 [126]. The role of HIF-2 may be underestimated, which may explain why trials of HIF-2 inhibitors in combination with TACE are relatively rare. A recent study [127] utilized a multifunctional polyvinyl alcohol (PVA)/hyaluronic acid (HA)-based microsphere (PT/DOX-MS) loaded with doxorubicin and PT-2385, a potent HIF-2α inhibitor, to improve the treatment of HCC. The results showed that PT/DOX-MS can block tumor cells in the G2/M phase. The introduction of PT-2385 effectively suppresses the expression levels of HIF-2α in hypoxic HCC cells, thereby downregulating the expression levels of Cyclin D1, VEGF, and TGF-α. Additionally, the combination of doxorubicin and PT-2385 can jointly inhibit the expression of VEGF. This suggests that HIF-2α may be an ideal target for TACE therapy.

**Table 1:** Summary of the therapeutic strategies of TACE combined with HIF-related pathway inhibitors for HCC.

HIF Isoform	Year	Refs.	Outcome	Targets Affected	Cancer Hallmark Affected
HIF-1	2023	[114]	Melatonin could inhibit tumor cell proliferation and migration by targeting HIF-1α and VEGF-A.	HIF-1α	↓HIF-1α ↓VEGF-A ↓MMP-2 ↓MMP-9 ↑E-cadherin
	2015	[121]	HIF-1α inhibitor LW6 attenuated the hypoxia-induced fibrosis progression in vivo. HIF-1α by HIF-1α-siRNA significantly decreased the expression of TGF-β1 and VEGF in hypoxic hepatocytes.	HIF-1α	↓HIF-1α ↓VEGF ↓TGF-β1 ↓Collagen I ↓α-SMA ↓Fibrosis
	2010	[115]	10-hydroxycamptothecin is a HIF-1α inhibitor.	HIF-1α	↓HIF-1α ↓VEGF ↓MVD
	2015	[116]	Liposomal curcumin could block HIF-1α-mediated angiogenesis.	HIF-1α	↓HIF-1α ↓VEGF ↓MVD
	2019	[117]	Curcumin liposome suppressed HIF-1α and survivin levels and inhibited the angiogenesis in VX2 rabbits after TAE.	HIF-1α	↓HIF-1α ↓VEGF ↓MVD ↓Survivin ↓Proliferation ↑Apoptosis
	2013	[119]	Ginsenoside Rg3 combined with TAE could effectively inhibit tumor growth by inhibiting tumor angiogenesis and inducing cancer cell apoptosis.	VEGF	↓VEGF ↓CD31 ↓Angiogenesis ↑Caspase-3 ↑Bax
	2016	[120]	The combination of ginsenoside Rg3 and TACE provided a greater survival benefit than TACE alone in patients with HCC.	VEGF	↑Overall survival ↑Time to progression ↑Time to untreatable progression ↑Disease control rate
HIF-2	2022	[127]	PT-2385 could effectively inhibit the expression level of HIF-2α in hypoxic HCC cells, thereby down-regulating the expression levels of Cyclin D1, VEGF and TGF-α.	HIF-2α	↓HIF-2α ↓VEGF ↓TGF-α ↓Cyclin D1

↓ = inhibit or reduce; ↑ = induce or increase.

### 6.2. Combination of Hypoxia-Activated Prodrugs and TACE

#### 6.2.1. Tirapazamine

Tirapazamine (TPZ), a bioreductive agent, is preferentially toxic to hypoxic cells. Under hypoxic conditions, TPZ is metabolized by an intracellular reductase to form a highly reactive radical species capable of inducing DNA single- and double-strand breaks and chromosome aberrations, resulting in cell death. In the presence of oxygen, the TPZ radical is oxidized back to the parent molecule, thereby largely preventing radical-induced damage [128]. In 2011, Sonoda et al. [129] found that the combination of intraperitoneal TPZ and TAE with gelatin microspheres significantly reduced the tumor growth rate compared with TAE or TPZ treatment alone in the rabbit VX2 model. Subsequently, another study by Lin et al. [130] reported that the combination of intravenous TPZ and hepatic arterial ligation had synergistic tumor-killing activity against hepatocellular carcinoma (HCC) in HBx transgenic mice. The safety findings of the toxicological study by Liu et al. [131] involving rats supported the clinical usage of the intraarterial injection of TPZ in combination with embolization. Several follow-up Phase I trials [132,133] demonstrated the safety and tolerability of intraarterial TPZ with TAE/TACE for HCC, yielding promising tumor responses. Li et al. [8] prepared TPZ-loaded CalliSpheres microspheres (CSMTPZs) and found that CSMTPZ therapy exhibited advantages in terms of hypoxia-selected cytotoxicity, tumor apoptosis and necrosis, animal survival, and safety over the conventional combination of TPZ and TAE in the rabbit VX2 model.

#### 6.2.2. TH-302

TH-302 (evofosfamide) is a 2-nitroimidazole-triggered HAP of the cytotoxin bromo-isophosphoramide mustard [134]. The dinitroimidazole structure is fragmented with an alkylating agent, dibromoisophosphoramide mustard, that selectively binds to the DNA and kills the tumor cells. Thus, it exerts little activity in the normoxic zone and has fewer side effects on normal tissues. TH-302 has shown broad-spectrum anticancer efficacy in multiple human cell lines and xenograft models [88,135,136,137,138,139]. In the rabbit VX2 model, Duran et al. [140] combined the conventional TACE and TH-302 by mixing the doxorubicin/Lipiodol emulsion and TH-302, followed by embolization with 100–300 μm bland beads. The results indicate that conventional TACE+TH-302 induced smaller tumor volumes, lower tumor growth rates, higher necrotic fractions, and exhibited no additional toxicity profile compared to conventional TACE. Another rabbit VX2 model by Ma et al. [141] involved the preparation of TH-302-loaded poly (lactic-co-glycolic acid) (PLGA)-based TACE microspheres. The results demonstrated that the TH-302 loaded microspheres exhibited sustained drug release in the liver tissue and superior anti-tumor efficacy in comparison to TH-302 injection and TH-302+lipiodol. Furthermore, no significant toxicity was observed throughout the treatment period.

**Table 2:** Summary of the therapeutic strategies of TACE combined with hypoxia-activated prodrugs for HCC.

Compound	Year	Refs.	Outcome	Targets Affected	Cancer Hallmark Affected
Tirapazamine	2011	[129]	The combination of TPZ i.p. and gelatin microspheres (GMS) i.a. enhanced the antitumor effect of TPZ.	Hypoxic tumor	↓Tumor growth
	2016	[130]	At levels below the threshold oxygen levels created by hepatic artery ligation (HAL), TPZ was activated and killed the hypoxic cells, but spared the normoxic cells.	Hypoxic tumor	↑Necrosis ↑Apoptosis
	2021	[131]	The safety findings of this toxicological study involving rats supported the clinical usage of the IA injection of TPZ in combination with embolization.	Hypoxic tumor	ALT Total bilirubin Histopathology
	2021	[133]	TPZ may be synergistic with TAE.	Hypoxic tumor	Tumor responses were evaluated using mRECIST criteria
	2022	[132]	TPZ i.a., in combination with TAE, was well tolerated and showed promising efficacy signals in intermediate-stage HCC.	Hypoxic tumor	Tumor responses were evaluated using mRECIST criteria
	2022	[8]	TPZ may exert synergistic tumor-killing activity with TAE for liver cancer.	Hypoxic tumor	↑Necrosis ↑Apoptosis
TH-302	2017	[140]	Evofosfamide in combination with conventional TACE enhanced anticancer effects.	Hypoxic tumor	↓Ki-67 ↑γ-H2A.X ↑Annexin V ↑Caspase-3 ↑Apoptosis
	2020	[141]	TH-302 is a hypoxia-activated prodrug targeting the intra-tumoral hypoxic environment.	Hypoxic tumor	↑Necrosis ↑Apoptosis

↓ = inhibit or reduce; ↑ = induce or increase.

### 6.3. Combination of Gene Therapy and TACE

#### 6.3.1. Hypoxia Pathway-Related Gene Therapy

RNA modulation is a common modality for HIF-related gene therapy. Previous studies have proposed the use of the RNA interference (RNAi) of HIF-1α to enhance the efficacy of TAE in the treatment of HCC. RNAi is a process whereby the expression of specific genes is silenced by the introduction of small interfering RNAs (siRNAs), endogenous microRNAs (miRs), and other short double-stranded RNAs [142]. The study by Chen et al. [143] confirmed that RNAi of HIF-1α improves the efficacy of TAE in the treatment of HCC. Its resulting HIF-1α silencing effectively inhibits the increase in VEGF expression and MVD after TAE, inhibits liver tumor growth, and reduces the number of lung metastases. The results of their subsequent study [144], which employed ultrasound-guided HIF-1α RNAi, demonstrated an improvement in the efficacy of TACE in the treatment of HCC, thereby further confirming previous findings. Moreover, the study by Ni et al. [145] yielded comparable outcomes. In this study, the iodized oil emulsion was prepared by combining lipiodol with a siRNA transfection compound, which was then delivered via the hepatic artery during the TAE procedure. In the rabbit VX2 model by Guo et al. [146], the TAE with drug-free microspheres combined with intraarterial transfection of HIF-1α shRNA on HCC demonstrated superior anti-tumor efficacy compared to monotherapy.

The combination of TACE with HIF-related gene knockdown represents a promising therapeutic approach for the treatment of HCC. Liu et al. [147] found that the myocardial infarction-associated transcript (MIAT)/miR-203a/HIF-1α axis could affect the efficacy of TAE. MIAT and HIF-1α were highly expressed, and miR-203a was lowly expressed in hypoxia-stimulated hepatocellular carcinoma cells after TACE. The MIAT gene regulated the miR-203a/HIF-1α axis, and MIAT knockdown enhanced TAE-mediated antitumor effects by upregulating miR-203a and downregulating HIF-1α. In addition, the lentiviral delivery (LV-H721) of the CRISPR/Cas9 protein and an HIF-1α-specific small guide RNA (sgRNA) resulted in highly efficient HIF-1α modification. One study employed the CRISPR/Cas9-mediated knockdown of HIF-1α [148]. The results demonstrated that the lentiviral delivery of CRISPR/Cas9 protein and HIF-1α-specific sgRNA was an effective method for modifying HIF-1α. Furthermore, the combination of CRISPR/Cas9-mediated HIF-1α knockdown and TAE was found to significantly suppress tumors and prolong the survival time of HCC mice.

#### 6.3.2. Hypoxia-Targeted Oncolytic Virus

In 2008, Altomonte et al. [149] proposed a novel method of treatment for HCC in rats, termed viroembolization. This approach involved the co-administration of recombinant vesicular stomatitis virus (VSV) with degradable starch microspheres (DSM), which were injected through the hepatic artery. The researchers observed that viral embolization induced apoptosis in tumor margins that survived embolization, significantly reducing intratumoral CD31 staining. Additionally, the procedure prevented neointimal formation after embolization, recruited NK cells and CD8+ T cells for infiltration, and led to massive tumor necrosis. The study by Sun et al. [150] demonstrated, for the first time, that the portal infusion of adeno-associated viral vectors expressing antisense HIF1-α downregulated HIF-1α and its downstream effectors, including VEGF, glucose transporter 1 (GLUT1), and LDHA, and enhanced the inhibitory effect of TAE on the growth of HCC in rats. Zhang et al. [151] synthesized a hypoxia-replicative oncolytic adenovirus (HYAD) and constructed VX2 HCC rabbit models by HYAD perfusion combined with PVA embolization. The results showed that HYAD was expressed and replicated in the presence of HIF-1α expression or hypoxia in in vitro experiments. In the in vivo experiments in the VX2 model, HYAD perfusion combined with PVA embolization resulted in the highest expression and the longest expression duration compared with HYAD perfusion alone, wild adenovirus type 5 (WT) perfusion combined with PVA embolization, and WT perfusion alone.

In another aspect, data from a rabbit VX2 tumor model found transarterial viroembolization (TAVE) to be the most effective modality, with more homogeneous oncolytic virus distribution and therapeutic efficacy compared to other delivery methods (intratumoral injection and intravenous injection). TAVE is the optimal and safe therapy for the treatment of immune-refractory HCC, and the synergistic effect achieves significant tumor response, standby effect, survival benefit, and anti-tumor immune memory, providing an innovative therapeutic approach for clinical practice [152]. It has to be acknowledged that the combination of TACE and oncolytic virus shows great promise, whether targeting hypoxic tumor cells or not.

**Table 3:** Summary of the therapeutic strategies of TACE combined with gene therapy for HCC.

Therapy Type	Year	Refs.	Outcome	Targets Affected	Cancer Hallmark Affected
Hypoxia pathway-related gene therapy	2012	[143]	HIF-1α RNAi visibly reduced the expression of HIF-1α and VEGF, suppressed tumor angiogenesis, and attenuated metastasis.	HIF-1α	↓HIF-1α ↓VEGF ↓MVD
	2015	[144]	HIF-1α RNAi could downregulate the levels of HIF-1α and VEGF, inhibit tumor angiogenesis, and lessen metastases.	HIF-1α	↓HIF-1α ↓VEGF ↓MVD
	2017	[145]	HIF-1α-siRNA could inhibit the expression levels of HIF-1α and VEGF effectively.	HIF-1α	↓HIF-1α ↓VEGF
	2020	[146]	HIF-1α shRNA could decrease the formation of blood vessels, slow tumor growth, reduce tumor size, and promote tumor cell apoptosis.	HIF-1α	↓HIF-1α ↓VEGF ↓CD34
	2020	[147]	MIAT knockdown potentiated the therapeutic effect of TAE in liver cancer by regulating the miR-203a/HIF-1α axis in vitro and in vivo.	MIAT/ miR-203a/HIF-1α	↑miR-203a ↓HIF-1α
	2018	[148]	The combination of CRISPR/Cas9-mediated HIF-1α knockdown and TAE was found to significantly suppress tumors.	HIF-1α	↓HIF-1α ↓CD31 ↓Invasiveness ↓Migration ↓Proliferation ↑Apoptosis
Hypoxia-targeted oncolytic virus	2009	[150]	Intraportal delivery of adeno-associated viral vectors expressing antisense HIF-α augmented TAE to combat hepatocellular carcinoma.	HIF-1α	↓HIF-1α ↓VEGF ↓GLUT1 ↓LDHA ↓Proliferation ↑Apoptosis
	2019	[151]	Adenovirus expression protein E1A has the properties of promoting apoptosis, inhibiting invasion, and inhibiting metastasis.	Hypoxic tumor	↓Proliferation ↓Migration ↑Apoptosis

↓ = inhibit or reduce; ↑ = induce or increase.

## 7. Conclusions

In view of the negative impact of local hypoxia on the therapeutic efficacy of TACE for HCC, hypoxia represents a promising target for combination use with TACE. Previous clinical phase III trials on TPZ for solid tumors showed negative results, which is likely due to the fact that the hypoxia within tumors is not sufficient to effectively activate TPZ to exert its hypoxia-selective cytotoxicity. In contrast, TACE generates a sufficient hypoxic microenvironment, which is conducive to the effectiveness of TPZ. Encouraging results have been observed in the phase I trial investigating TPZ with TAE for HCC, and phase II trials are currently underway. Future directions in this field include a comprehensive investigation of the role of possible interactions between hypoxia and the effect of TAE during the TACE procedure. Additionally, there is a need to develop new formulations of hypoxia-targeted drugs suitable for combination with TACE, such as novel HAPs that can be suspended in lipiodol or loaded onto drug-eluting microspheres at high doses. Moreover, nanotechnology has been utilized to deliver anti-tumor drugs and embolize tumor blood vessels. Further studies are required to assess the feasibility of the use of nanomaterials to deliver hypoxia-targeted drugs and their efficacy and safety as a chemoembolization agent targeting hypoxic tumor cells. Overall, the combination of TACE with hypoxia-targeted therapy constitutes an effective strategy, and continued investigation and innovation in this field will be crucial to improving outcomes for patients with HCC.

## Data Availability

Data sharing is not applicable to this article.
